# The Reliability and Validity of the Loadsol^®^ under Various Walking and Running Conditions

**DOI:** 10.3390/s19020265

**Published:** 2019-01-11

**Authors:** Kristen E. Renner, DS Blaise Williams, Robin M. Queen

**Affiliations:** 1 Kevin P. Granata Biomechanics Lab, Biomedical Engineering and Mechanics, Virginia Tech, Blacksburg, VA 24061, USA; rmqueen@vt.edu; 2 Nike Sport Research Lab, Nike, INC, Beaverton, OR, 97005, USA; blaise.williams@icloud.com; 3 Department of Orthopaedic Surgery, Virginia Tech Carilion School of Medicine, Roanoke, VA 24016, USA

**Keywords:** gait mechanics, ground reaction force, reliability, Bland–Altman, in-shoe

## Abstract

The assessment of loading during walking and running has historically been limited to data collection in laboratory settings or with devices that require a computer connection. This study aims to determine if the loadsol^®^—a single sensor wireless insole—is a valid and reliable method of assessing force. Thirty (17 male and 13 female) recreationally active individuals were recruited for a two visit study where they walked (1.3 m/s) and ran (3.0 and 3.5 m/s) at a 0%, 10% incline, and 10% decline, with the visits approximately one week apart. Ground reaction force data was collected on an instrumented treadmill (1440 Hz) and with the loadsol^®^ (100 Hz). Ten individuals completed the day 1 protocol with a newer 200 Hz loadsol^®^. Intraclass correlation coefficients (ICC3,k) were used to assess validity and reliability and Bland–Altman plots were generated to better understand loadsol^®^ validity. Across conditions, the peak force ICCs ranged from 0.78 to 0.97, which increased to 0.84–0.99 with the 200 Hz insoles. Similarly, the loading rate ICCs improved from 0.61 to 0.97 to 0.80–0.96 and impulse improved from 0.61 to 0.97 to 0.90–0.97. The 200 Hz insoles may be needed for loading rate and impulse in running. For both walking and running, the loadsol^®^ has excellent between-day reliability (>0.76).

## 1. Introduction

Ground reaction forces are often used in the assessment of both normal and pathologic gait as a means of understanding various loading parameters [1,2,3,4,5,6]. Historically, studies focusing on gait biomechanics in which there was a desire to assess lower extremity loading have been limited to research facilities with either force plates, pressure pads, or an instrumented treadmill [7,8,9,10,11,12,13]. Over the last decade, a number of devices have been developed that allow for the assessment of force measures through in-shoe pressure insoles. The development of these devices has allowed researchers and clinicians to assess loading parameters in walking [14,15,16], running [17,18], jumping [18], cutting, and agility [18,19].

Systems like the pedar^®^-X (Novel Electronics, St. Paul, MN, USA) and the F-Scan (Tekscan, Inc, South Boston, MA, USA) allow for the collection of in-shoe pressure data outside of traditional laboratory systems. However, these systems both require cabling from the insoles to a controller that the research participant has to carry, typically in a pouch or backpack [14,20]. The development of in-shoe pressure sensors like these has expanded the breadth of studies that can be conducted during various activities of daily living and athletic movements. However, there are some limitations in both clinical and rehabilitation settings. In-shoe pressure measurement devices are expensive and therefore may be impractical for many clinics [21]. Additionally, because of the wires that run from the insoles to the waist pouch or small backpack, there are concerns that the equipment can alter locomotion and change participant behavior. Based on these concerns, there has been an interest in the development of sensor technology that would allow for the collection of wireless in-shoe force data.

**Figure 1:**
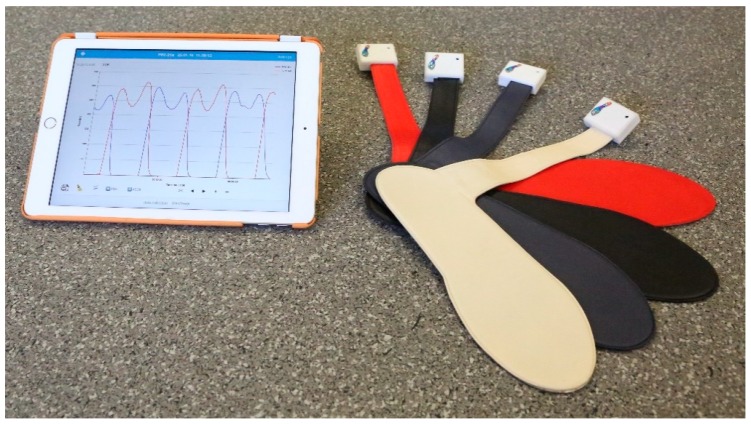
The loadsol^®^ and a sample data file are pictured here. Photo taken by Michael Diersing.

Recently, a single sensor insole was developed by Novel Electronics (St. Paul, MN, USA), which allows for the collection of in-shoe force data without cabling. The loadsol^®^ is a thin, single sensor insole that measures the normal force between the foot and the shoe. This device has a small, flat cable that comes out of the shoe and has an electronics box on the end that allows for the transmission of the data to a smartphone or tablet in real-time (Figure 1). This small box can be clipped to the shoelaces or on the tongue of the shoe to decrease any interference with participants’ movement; the position of the box is advantageous because it will not impact the movement patterns of participants. The data is stored on the mobile device and can be exported as a text file for additional analysis as desired. Given the size and capabilities of the loadsol^®^, the system has the potential to be used for a variety of clinical and in-the-field applications. However, before the loadsol^®^ can be used for these applications, it is imperative that the device’s reliability and the validity be established.

For a better understanding of the applicability of the loadsol^®^, the reliability and validity of various load parameters such as peak force, loading rate, and impulse during various tasks (inclined, declined, and level walking and running) should be established. Loading rate is the rate that force increases as the foot strikes the ground in walking or running; impulse is the area under the force–time curve for a single step. Thus far, only one study has published data concerning the validity and reliability of the loadsol^®^ during walking and/or running [22]. This study investigated the validity and reliability of the loadsol^®^ during 1.4 m/s walking and 2.8 m/s running on a flat treadmill. Burns et al. determined that the loadsol^®^ had excellent agreement and between visit reliability for the peak ground reaction force and impulse measurements [22]. This article did not examine inclined or declined conditions or report loading rate which may be more sensitive to the decreased sampling frequency of the loadsol^®^. Previous work has shown differences in plantar loading and peak vertical ground reaction force (vGRF) between various levels of inclined and declined walking has been reported using the pedar^®^ system, indicating that it is important to understand reliability and validity during inclined and declined conditions as well as during level walking [23,24]. Additionally, Peebles et al. found that the loadsol^®^ sampling rate can impact the validity and reliability of the loadsol^®^ during dynamic movements. It was found that stop jump and single hop force data collected with the 200 Hz insoles had improved validity when compared with the 100 Hz data [25]. It is unknown if a higher sampling rate is needed during less dynamic activities like walking and running.

Therefore, the purpose of this study was to determine the between-day reliability as well as the validity of the loadsol^®^ compared to an instrumented treadmill during walking and running at various speeds and inclines. Based on what is currently known about the reliability and validity of the in-shoe pressure systems, we hypothesized that the peak force, the impulse, and the loading rate from the loadsol^®^ would demonstrate good to excellent agreement with the corresponding force plate data. Further, we hypothesized that for each variable of interest, the loadsol^®^ would demonstrate good to excellent between-day reliability.

## 2. Materials and Methods

In order to complete this project, 30 recreationally active, healthy young adults (17 male and 13 female) between the ages of 18 and 30 were recruited and asked to complete this two-visit study, Table 1. All participants were comfortable walking on a treadmill and had previous treadmill experience. Recreationally active was defined as exercising at least 3 times a week for 30 min or more each session. Based on previous literature, gender does not alter validity and reliability, therefore, the impact of gender was not investigated in this study [15,16,17]. All study participants signed an Institutional Review Board approved consent form prior to study initiation. To be included in this study, participants had previous treadmill experience and could not have any musculoskeletal diseases or injuries that prevented normal activity for more than 2 days in the last 3 months.

Each participant was provided with a pair of neutral running shoes—Nike Air Pegasus (Nike Inc., Beaverton, OR, USA)—and the appropriate size loadsol^®^ pair at the beginning of the session (Figure 1). The loadsol^®^ is a single capacitive force sensor along the length of the insole. The primary advantage of the loadsol^®^ is the ability to collect and store data via Bluetooth without cables [25]. The participants’ age, height, and weight were recorded. After a 5 min warm up, the weight of the participant was entered in Newtons (N) into the loadsol^®^ application on an iPad (Apple Inc., Cupertino, CA, USA) and the resolution was set to 5 N to allow for a force range of 0 to 2550 N. The resolution indicates the increments over which the loadsol^®^ collects data. In this study, data was collected in 5 N increments for the range of 0 to 2550 N. If the load is expected to exceed 2550 N the resolution should be changed to 10 N. The loadsol^®^ was calibrated using the procedure previously described by Peebles et al. [25]. Participants were instructed to load the insoles with their full bodyweight in a single-leg stance and then unloading the insole three times on each foot following the company’s calibration procedures. The calibration was saved and was checked to ensure that the load during single leg stance was within 5% of the body mass that was entered into the loadsol^®^ application. If the calibration was not acceptable, the calibration procedure was repeated.

Following calibration, each participant completed a total of 9 trials. One walking speed—1.3 m/s—and two running speeds—3.0 and 3.5 m/s—were selected. Each speed was performed at three grades of incline (0% level walking, 10% incline, and 10% decline). The order of these 9 conditions were randomized using a random sequence generator for each visit for each participant. Participants were encouraged to take brakes as needed. Each trial lasted 1 min, and data was collected for the middle 30 s using the loadsol^®^ (100 Hz) and two force plates within a fore–aft split belt, instrumented treadmill (Compact Tandem Force-Sensing Treadmill, Model: DBCEEWI, AMTI, Watertown, MA, USA) (1440 Hz). This protocol was repeated approximately one week later for each participant to assess the between-day reliability of the loadsol^®^.

**Figure 2:**
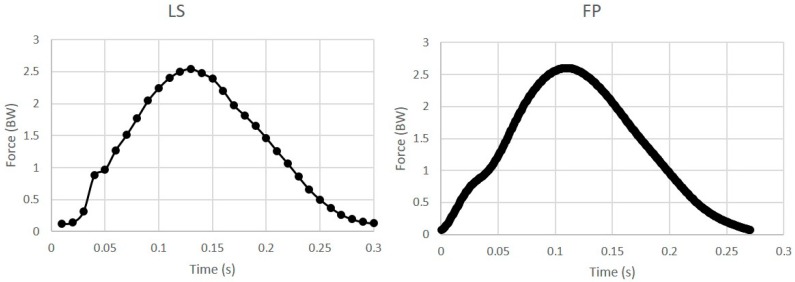
A representative image of the loadsol^®^ (**left**) and force plate (**right**) data for a single running step. Each dot represents one data point collected at 100 Hz for the loadsol^®^ and 1440 Hz for the force plate.

For this study only the left foot was analyzed and there was an average of 25.8 ± 4.8 steps at 1.3 m/s, 36.2 ± 12.7 steps at 3.0 m/s, and 35.7 ± 13.6 steps at 3.5 m/s. The force plate vGRF was filtered in Matlab (version 9.2.0, Mathworks, Natick, MA, USA) using a 4th order recursive Butterworth filter with a cutoff frequency of 25 Hz [26]; the loadsol^®^ GRF data used in the analysis was not filtered (Figure 2). The decision to not filter the loadsol^®^ data was based on a preliminary analysis comparing the variables of interest before and after filtering the data. The cutoffs selected for this preliminary analysis were based on a frequency analysis of the loadsol^®^ data which indicated that a 10 Hz and 20 Hz cutoff should be used for the data collected at 100 Hz and 200 Hz, respectively. Using a Butterworth filter with the above cutoffs did not impact the variables of interest and we therefore proceeded with the raw loadsol^®^ data. The variables of interest—peak weight acceptance force, impulse, and loading rate—were calculated for each step in MATLAB for both the force plate and loadsol^®^ data. Impulse was calculated as the area under the force–time curve from heel strike to toe-off, and loading rate was calculated as the slope of the weight acceptance portion of the force–time curve using the method presented by Goss and Gross [27]. These variables were calculated for each step and then averaged for the loadsol^®^ and the force plate data independently.

SPSS (version 25, IBM Analytics, Armonk, NY, USA) was used to run a paired *t*-test between the loadsol^®^ and force plate values on day 1 (α = 0.05). The validity of the loadsol^®^ was determined by calculating the intraclass correlation coefficient, ICC(3,k) with a 95% confidence interval, between the force plate and the loadsol^®^ using the data from day 1. Bland–Altman plots were also generated to look at the validity of the loadsol^®^ compared to the force plates. The between-day reliability was determined by calculating the ICC(3,k) between day 1 and day 2 for both the loadsol^®^ and force plate data in combination with Bland–Altman plots generated in MATLAB. The standard error of the mean (SEM) was calculated with the 95% confidence intervals for both the force plate and loadsol^®^. In order to categorize the ICC values for both the validity and reliability, the following categorization scheme was used; >0.75 is excellent, 0.60–0.74 is good, 0.40–0.59 is fair, and <0.40 is poor [28].

After the initial study was completed, a new 200 Hz loadsol^®^ was released. We repeated the day 1 testing and data processing protocol to determine if increasing the sampling rate to 200 Hz would improve the validity of the loadsol^®^ during both walking and running. We recruited 10 recreationally active athletes, Table 1, and completed the day 1 data collection procedures with a new randomized collection order as well as the same statistical analysis.

## 3. Results

**Figure 3:**
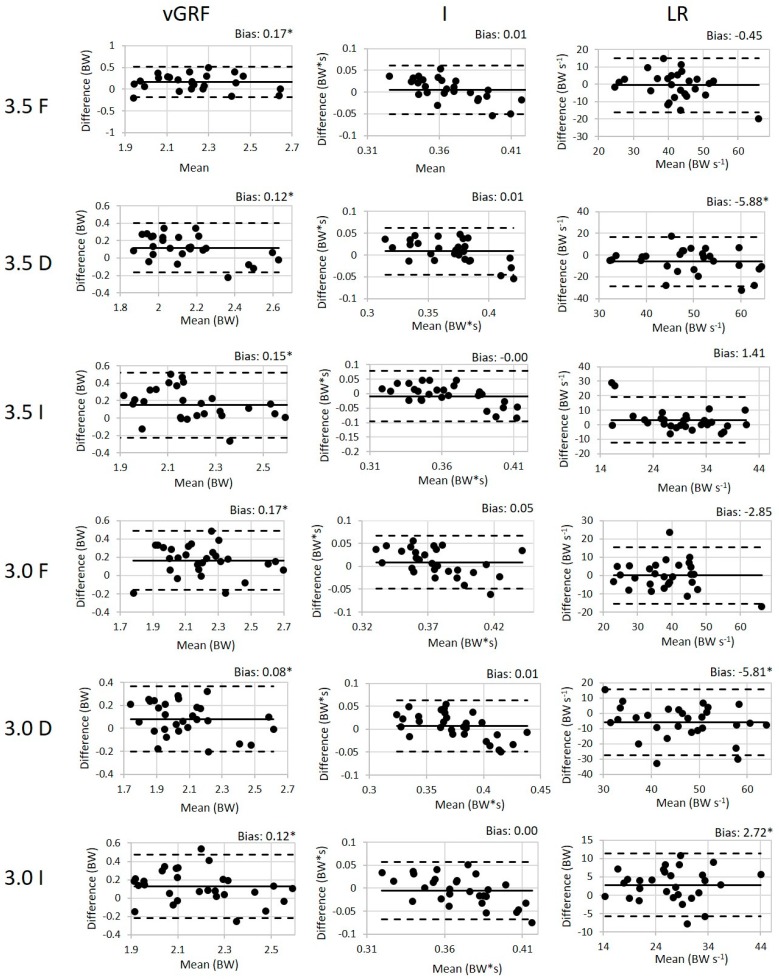
The Bland–Altman plots for peak force, impulse, and loading rate with the 100 Hz loadsol^®^ for both running speeds (3.0 and 3.5 m/s) flat (F), inclined (I), and declined (D). The difference is calculated by subtracting the loadsol^®^ values from the force plate values. The bias (mean difference) is displayed for each variable with a * indicating a statistically significant difference between the force plate and loadsol^®^.

**Figure 4:**
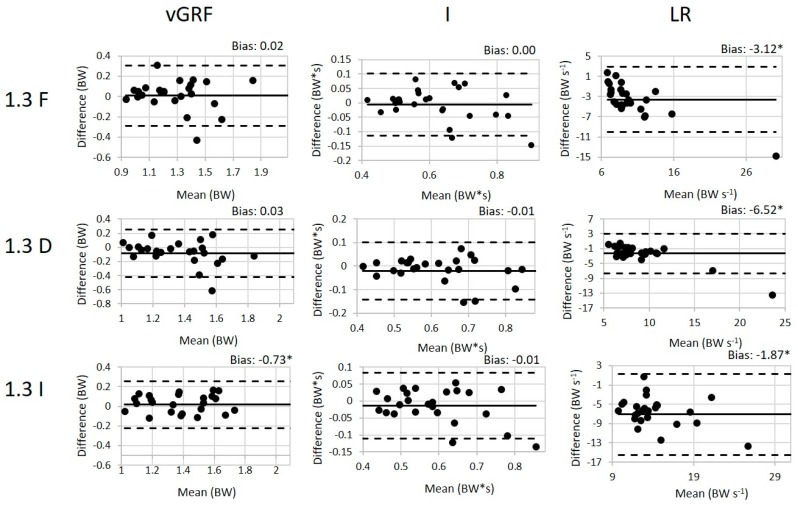
The Bland–Altman plots for each variable during walking conditions (1.3 m/s, flat (F), inclined (I), and declined (D)) with the 100 Hz loadsol^®^. The difference is calculated by subtracting the loadsol^®^ values from the force plate values. The bias (mean difference) is displayed for each variable with a * indicating a statistically significant difference between the force plate and loadsol^®^.

One subject was excluded from the 100 Hz analysis due to equipment challenges during data collection. For the remaining subjects, the values (mean ± SD) of the peak weight acceptance force, impulse (I), and loading rate (LR) for all conditions are presented in Table 2 along with the validity ICC results and the SEM between the loadsol^®^ and force plates. The walking ICC values for all variables of interest are good to excellent indicating that there is a good to excellent association between the loadsol^®^ and the force plates during walking. Similarly, the ICC values for the variables of interest during the running conditions ranged from 0.61 to 0.97 also indicating a good to excellent association between the loadsol^®^ and force plates during running. The paired *t*-tests indicated that there is a difference between methods in all running conditions for peak load and for the LR in the declined conditions and in the 3.0 m/s inclined condition. The Bland–Altman plots for all 100 Hz conditions are presented in Figure 3 and Figure 4.

**Figure 5:**
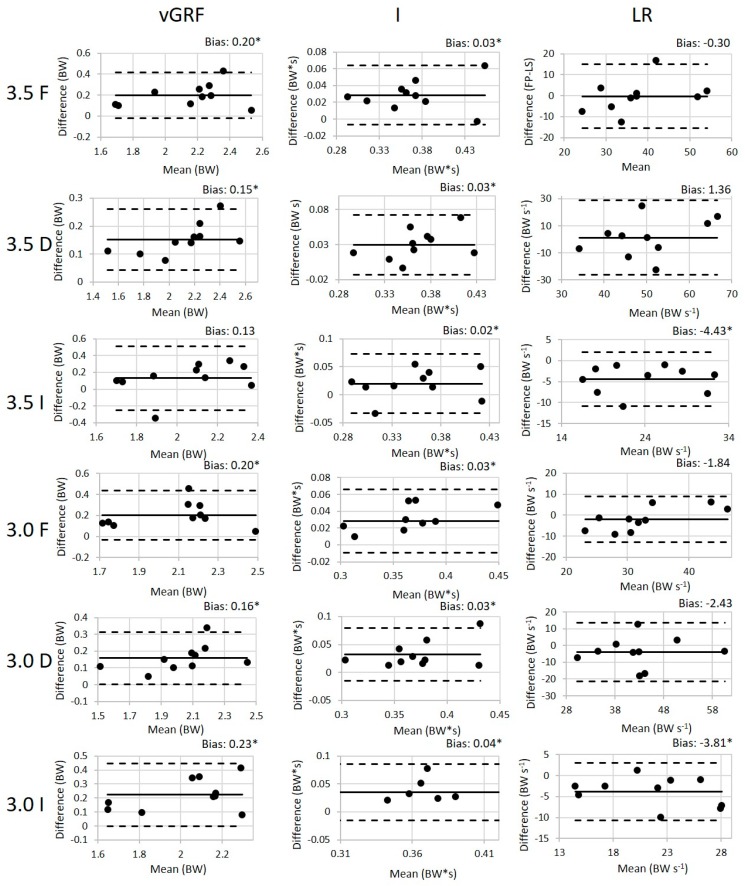
The Bland–Altman plots for the running conditions with the 200 Hz loadsol^®^ for both running speeds (3.0 and 3.5 m/s) flat (F), inclined (I), and declined (D). The difference is calculated by subtracting the loadsol^®^ values from the force plate values. The bias is displayed for each variable with a * indicating a statistically significant difference between the force plate and loadsol^®^.

**Figure 6:**
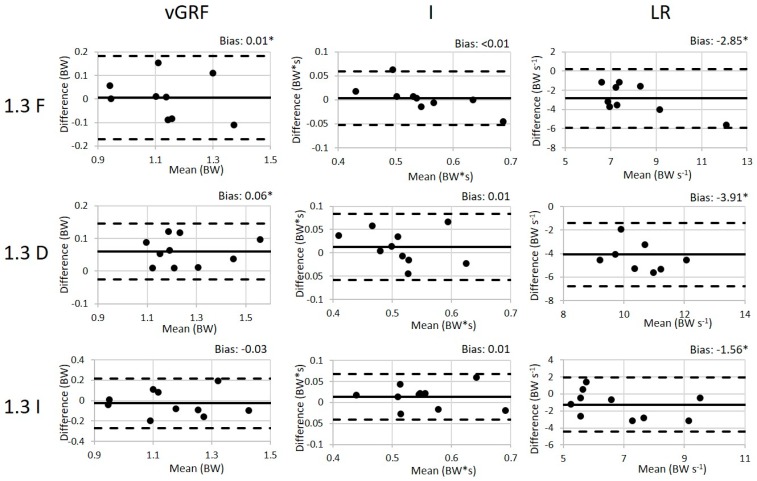
The Bland–Altman plots for walking conditions (1.3 m/s, flat (F), inclined (I), and declined (D)) with the 200 Hz loadsol^®^. The difference is calculated by subtracting the loadsol^®^ values from the force plate values. The bias is displayed for each variable with a * indicating a statistically significant difference between the force plate and loadsol^®^.

The 200 Hz validity results, Table 3, indicated excellent validity for peak load, impulse, and LR across all of the conditions. From the paired *t*-tests, all of the running conditions except for the 3.5 m/s incline had differences in peak load, all of the conditions had differences in impulse and the inclined conditions had differences in LR. The 200 Hz Bland–Altman plots support the good–excellent validity, Figure 5 and Figure 6.

For between-day reliability, the loadsol^®^ ICC values range from 0.90 to 0.99 for peak weight acceptance, 0.86–0.98 for impulse, and 0.72–0.99 for loading rate, Table 4. Peak force and impulse were all classified as having excellent reliability while loading rate ranges from good to excellent.

## 4. Discussion

The high ICC values presented in this study support our hypothesis that the loadsol^®^ is a valid and reliable method of collecting peak weight acceptance force, loading rate and impulse during level, inclined and declined walking and running. In addition to having high ICC values between the force plates and loadsol^®^, the bias for each variable is relatively small and most of the data falls within the 95% limits of agreement, which supports the loadsol^®^ being a valid and reliable system to assess load metrics during walking outside of the laboratory setting.

There is a significant bias in the peak load between the force plate and loadsol^®^. Generally, the loadsol^®^ is underestimating the force plate for the peak force and impulse measurements. This difference was expected based on previous literature that has discussed the impact of an insole on measured force values [25,29,30,31]. The loadsol^®^ overestimated the loading rate which differs from previously reported literature. Peebles et al. reported that the loadsol^®^ overestimated the loading rate for a stop jump, but underestimated for the single hop [25]. Seiberl et al. indicated that the loadsol^®^ overestimates the loading rate in running, but there are several methodological differences making direct comparisons of the findings between these studies difficult [31]. Seiberl et al. resampled the loadsol^®^ data from 100 Hz to 1000 Hz and used a different method of calculating the loading rate: the maximal slope of the increasing force during the impact phase [31]. However, when the same sample rate and calculations are utilized the loadsol^®^ appears to consistently overestimate the loading rate [25]. This is most likely due to the differences in sampling rates between the loadsol^®^ and the force plates.

Across all conditions, the 100 Hz loadsol^®^ has excellent validity and reliability, and the 200 Hz loadsol^®^ further improves the validity ICC values. The validity of the impulse varies greatly between walking and running—in walking the 100 Hz impulse is greater than 0.95, but in the running conditions it ranges from 0.61 to 0.97. We believe this could be the result of the lower sampling frequency, because the 200 Hz insoles improve the ICC values to all be greater than 0.90. Loading rate also has a wider range of ICC values (0.61–0.97) with the 100 Hz insoles. Similarly to the impulse measure, when the sampling frequency is increased to 200 Hz the ICC range is 0.80 to 0.96 moving all of the conditions into the excellent ICC range. We believe that the lower ICC values for the loading rate at 100 Hz could be due to fewer points of the linear portion of the ground reaction force trace. The method used to calculate loading rate in this article calculates the slope between 3% and 12% of the stance time which may be impacted by the number of data points used in this calculations for the loadsol^®^.

With respect to the ICC values of the loadsol^®^, Burns et al. reported similar ICC values for peak load and impulse during flat walking [22]. During flat running at 2.7 m/s, Burns et al. reported higher peak load and similar impulse ICC values. The increased ICC for peak load during running may be explained by a slower running speed and a difference in methodology. Burns et al. calculated the ICC by including all of the steps from each research participant resulting in 8000 data points. We decided to use the average of the steps for each participant resulting in fewer data points. This method was chosen because it more closely reflects standard data analysis methods. Burns et al. did not report loading rate results.

There are several potential limitations to this study. We selected representative running and walking speeds for this study. It is possible that increasing or decreasing the speeds could impact the validity of the loadsol^®^; if a study is focused on speeds that are much faster than 3.5 m/s or slower than 1.3 m/s, we recommend completing a similar protocol to confirm the validity and reliability of the loadsol^®^. Additionally, future research should attempt to limit the number of Bluetooth signals near the loadsol^®^ as they can cause the loadsol^®^ signal to drop frames. Concerning the 200 Hz insoles, we did not tested the between day repeatability, but we do not anticipate that the results would differ from the 100 Hz reliability ICC values presented here as the increased sampling rate should only impact the validity of the loadsol^®^, not the between day repeatability. Finally, the sample size used for the 200 Hz validity analysis is small, future studies using the 200 Hz insoles may benefit by confirming the validity with a larger sample side.

The ICC values and Bland–Altman plots for the peak force, impulse, and loading rate between the loadsol^®^ and the instrumented treadmill forces indicate that the loadsol^®^ can be used to assess load based parameters during various walking and running tasks. Given that the loadsol^®^ is a valid and reliable method of data collection and is less expensive than traditional force monitoring methods, these insoles have the potential to be used in the assessment and tracking of various clinical populations recovering from injuries and surgeries. These insoles could also be used to assess changes in load parameters in the clinical setting to track recovery and restoration of symmetric loading following a variety of surgical and nonsurgical interventions. The loadsol^®^ can also be used when asking patients to maintain a partial weight bearing protocol following surgery through setting load thresholds within the mobile application that will alter the patient when he/she is placing more load on the foot than is recommended during recovery. In addition, the loadsol^®^ can be used for research to better improve injury prevention interventions as well as rehabilitation programs to improve health outcomes that could ultimately improve the quality of life for many patients. These applications will need to be assessed in future studies.

## Figures and Tables

**Table 1:** Demographics.

**100 Hz**	Gender	Male (*n* = 17)	Female (*n* = 13)
	Age	20.94 ± 2.41	21.38 ± 3.01
	Height (m)	1.79 ± 0.07	1.69 ± 5.34
	Weight (kg)	73.50 ± 13.7	64.64 ± 7.93
**200 Hz**	Gender	Male (*n* = 6)	Female (*n* = 4)
	Age	22.8 ± 2.04	24.8 ± 0.96
	Height (m)	1.75 ± 0.03	1.75 ± 0.03
	Weight (kg)	79.40 ± 8.76	67.92 ± 3.32

**Table 2:** Day 1 results of peak force, impulse, loading rate for the force plate (FP) and loadsol^®^ (LS) are presented along with the standard error of the mean (SEM) and the intraclass correlation coefficients (ICC) with their 95% confidence intervals (CI).

Speed		3.5 m/s			3.0 m/s			1.3 m/s		
% Grade		0%	−10%	10%	0%	−10%	10%	0%	−10%	10%
**Peak Force (BW)**	**FP**	2.34 ± 0.24	2.21 ± 0.18	2.28 ± 0.18	2.28 ± 0.23	2.12 ± 0.21	2.21 ± 0.31	1.29 ± 0.22	1.43 ± 0.28	1.31 ± 0.22
	**SEM**	0.05	0.04	0.03	0.04	0.04	0.04	0.04	0.06	0.04
	**95% CI**	[2.25, 2.44]	[2.13, 2.27]	[2.21, 2.35]	[2.19, 2.37]	[2.04, 2.19]	[2.17, 2.32]	[1.20, 1.38]	[1.32, 1.55]	[1.22, 1.40]
	**LS**	2.18 ± 0.25	2.09 ± 0.25	2.13 ± 0.26	2.12 ± 0.25	2.04 ± 0.26	2.08 ± 0.32	1.27 ± 0.24	1. 40 ± 0.27	1.38 ± 0.27
	**SEM**	0.05	0.05	0.05	0.05	0.05	0.04	0.05	0.05	0.05
	**95% CI**	[2.08, 2.27]	[1.99, 2.19]	[2.03, 2.23]	[2.02, 2.21]	[1.94, 2.13]	[2.03, 2.21]	[1.17, 1.37]	[1.23, 1.51]	[1.27, 1.49]
	***p*-value**	<0.01 *	<0.01 *	<0.01 *	<0.01 *	<0.01 *	<0.01 *	0.50	0.09	0.04 *
	**ICC**	0.86	0.88	0.78	0.86	0.89	0.92	0.89	0.97	0.87
	**95% CI**	[0.69, 0.93]	[74, 0.95]	[0.53, 0.90]	[0.70, 0.94]	[0.77, 0.95]	[0.83, 0.96]	[0.75, 0.95]	[0.93, 0.99]	[0.71, 0.94]
Impulse (BW *s)	**FP**	0.37 ± 0.02	0.37 ± 0.02	0.36 ± 0.02	0.38 ± 0.02	0.38 ± 0.03	0.39 ± 0.10	0.61 ± 0.11	0.57 ± 0.10	0.61 ± 0.11
	**SEM**	<0.01	<0.01	<0.01	<0.01	<0.01	<0.01	0.02	0.02	0.02
	**95% CI**	[0.37, 0.38]	[0.36, 0.38]	[0.35, 0.37]	[0.37, 0.39]	[0.37, 0.39]	[0.36, 0.38]	[0.56,0.65]	[0.53, 0.61]	[0.56, 0.66]
	**LS**	0.37 ± 0.04	0.36 ± 0.04	0.36 ± 0.04	0.37 ± 0.04	0.37 ± 0.04	0.39 ± 0.10	0.61 ± 0.12	0.58 ± 0.12	0.62 ± 0.13
	**SEM**	0.01	0.01	0.01	0.01	0.01	0.01	0.02	0.02	0.03
	**95% CI**	[0.35, 0.38]	[0.35, 0.38]	[0.35, 0.38]	[0.36, 0.39]	[0.36, 0.39]	[0.36, 0.39]	[0.56, 0.66]	[0.53, 0.62]	[0.57, 0.68]
	***p*-value**	0.34	0.12	0.58	0.12	0.22	0.46	0.92	0.33	0.20
	**ICC**	0.68	0.77	0.61	0.69	0.79	0.97	0.96	0.96	0.95
	**95% CI**	[0.30, 0.85]	[0.50, 0.90]	[0.16, 0.82]	[0.34, 0.86]	[0.55, 0.90]	[0.94, 0.99]	[0.90, 0.98]	[0.90, 0.98]	[0.88, 0.98]
**Loading Rate (BW/s)**	**FP**	41.91 ± 7.96	46.15 ± 9.39	30.87 ± 6.47	38.38 ± 9.16	43.34 ± 9.61	27.43 ± 8.55	7.82 ± 1.81	10.91 ± 3.39	7.50 ± 2.00
	**SEM**	1.50	1.81	1.24	1.73	1.79	1.31	0.36	0.68	0.40
	**95% CI**	[38.82, 44.99]	[42.43, 49.86]	[28.31,33.43]	[34.82, 41.39]	[39.68, 46.99]	[25.66, 31.03]	[7.08, 8.57]	[9.51, 12.31]	[6.68, 8.33]
	**LS**	42.35 ± 10.46	52.02 ± 12.25	29.46 ± 6.72	38.39 ± 10.68	49.14 ± 11.95	24.70 ± 8.15	10.95 ± 3.14	17.44 ± 4.49	9.37 ± 2.97
	**SEM**	1.98	2.36	1.29	2.02	2.22	1.28	0.63	0.90	0.59
	**95% CI**	[38.30, 46.41]	[47.18, 56.87]	[26.80, 2.11]	[34.24, 42.53]	[44.60, 53.69]	[22.89, 28.15]	[9.65, 12.24]	[15.58, 19.29]	[8.14, 10.60]
	***p*-value**	0.77	0.01 *	0.11	0.99	<0.01 *	<0.01 *	<0.01 *	<0.01 *	<0.01 *
	**ICC**	0.78	0.61	0.87	0.92	0.97	0.93	0.72	0.83	0.90
	**95% CI**	[0.53, 0.90]	[0.14, 0.82]	[0.72, 0.94]	[0.60, 0.91]	[0.26, 0.84]	[0.85, 0.97]	[0.36,0.88]	[0.60, 0.92]	[0.77, 0.95]

* Statistical difference between the loadsol^®^ and force plate values.

**Table 3:** Peak force, impulse, and loading rate (LR) results for the force plate (FP) and loadsol^®^ (LS) results of the 200 Hz loadsol^®^.

Speed		3.5 m/s			3.0 m/s			1.3 m/s		
% Grade		0%	−10%	10%	0%	−10%	10%	0%	−10%	10%
**Peak Force (BW)**	**FP**	2.24 ± 0.30	2.19 ± 0.32	2.12 ± 0.29	2.19 ± 0.27	2.11 ± 0.27	2.15 ± 0.27	1.14 ± 0.14	1.28 ± 0.15	1.15 ± 0.16
	**SEM**	0.09	0.10	0.09	0.09	0.09	0.09	0.05	0.05	0.05
	**95% CI**	[2.02, 2.45]	[1.96, 2.42]	[1.91, 2.32]	[1.99, 2.19]	[1.92, 2.30]	[1.95, 2.34]	[1.03, 1.24]	[1.17, 1.39]	[1.04, 1.27]
	**LS**	2.04 ± 0.26	2.04 ± 0.29	1.98 ± 0.22	1.98 ± 0.25	1.95 ± 0.23	1.92 ± 0.22	1.13 ± 0.16	1.22 ± 0.15	1.18 ± 0.17
	**SEM**	0.08	0.09	0.07	0.08	0.07	0.07	0.05	0.05	0.05
	**95% CI**	[1.85, 2.23]	[1.83, 2.24]	[1.83, 2.14]	[1.80, 2.16]	[1.79, 2.12]	[1.76, 2.08]	[1.01, 1.25]	[1.11, 1.33]	[1.06, 1.30]
	***p*-value**	<0.01 *	<0.01 *	0.06	<0.01 *	<0.01 *	<0.01 *	0.84	0.72	0.01 *
	**ICC**	0.96	0.99	0.84	0.95	0.97	0.94	0.90	0.98	0.84
	**95% CI**	[0.84, 0.99]	[0.97, 1.00]	[0.34, 0.96]	[0.78, 0.99]	[0.90, 1.00]	[0.78, 0.99]	[0.55, 0.98]	[0.91, 1.00]	[0.36, 0.96]
Impulse (BW *s)	**FP**	0.38 ± 0.05	0.38 ± 0.04	0.36 ± 0.05	0.39 ± 0.05	0.39 ± 0.04	0.38 ± 0.05	0.55 ± 0.07	0.52 ± 0.06	0.56 ± 0.07
	**SEM**	0.02	0.01	0.02	0.02	0.01	0.01	0.02	0.02	0.02
	**95% CI**	[0.35, 0.42]	[0.35, 0.41]	[0.33, 0.40]	[0.35, 0.42]	[0.36, 0.42]	[0.35, 0.42]	[0.50, 0.60]	[0.48, 0.56]	[0.51, 0.61]
	**LS**	0.36 ± 0.05	0.35 ± 0.03	0.34 ± 0.04	0.36 ± 0.05	0.36 ± 0.03	0.35 ± 0.04	0.55 ± 0.09	0.51 ± 0.07	0.55 ± 0.07
	**SEM**	0.02	0.01	0.01	0.02	0.01	0.01	0.03	0.02	0.02
	**95% CI**	[0.32, 0.39]	[0.33, 0.38]	[0.31, 0.38]	[0.32, 0.40]	[0.33, 0.38]	[0.32, 0.38]	[0.48, 0.61]	[0.46, 0.56]	[0.49, 0.60]
	***p*-value**	<0.01 *	<0.01 *	0.05 *	<0.01 *	<0.01 *	<0.01 *	<0.01 *	0.31	<0.01 *
	**ICC**	0.97	0.91	0.91	0.96	0.90	0.92	0.97	0.91	0.96
	**95% CI**	[0.87, 0.99]	[0.66, 0.98]	[0.65, 0.98]	[0.85, 0.99]	[0.60, 0.98]	[0.67, 0.98]	[0.84, 0.99]	[0.65, 0.98]	[0.85, 0.99]
**Loading Rate (BW/s)**	**FP**	37.50 ± 11.54	50.55 ± 15.08	21.56 ± 6.01	31.67 ± 9.54	41.66 ± 10.11	19.76 ± 4.74	6.56 ± 1.32	10.05 ± 4.02	6.13 ± 1.52
	**SEM**	3.65	5.03	1.90	3.02	3.37	1.50	0.44	1.27	0.51
	**95% CI**	[29.24, 45.75]	[37.94, 63.16]	[17.27, 25.86]	[24.85, 38.49]	[33.89, 49.44]	[16.37, 23.14]	[5.54, 7.58]	[7.17, 12.93]	[4.96, 7.31]
	**LS**	37.807 ± 8.75	49.19 ± 8.43	26.00 ± 5.85	33.51 ± 5.71	44.90 ± 9.09	23.58 ± 5.80	9.41 ± 2.34	13.95 ± 3.37	7.70 ± 1.93
	**SEM**	2.77	2.98	1.85	1.81	3.09	1.83	0.78	1.07	0.65
	**95% CI**	[31.54, 44.06]	[42.14, 56.24]	[21.81, 30.18]	[29.43, 37.60]	[39.98, 51.22]	[19.43, 27.72]	[7.61, 11.20]	[11.54, 16.37]	[6.21, 9.19]
	***p*-value**	0.91	0.71	<0.01 *	0.32	0.38	<0.01 *	0.52	0.16	0.01 *
	**ICC**	0.83	0.80	0.92	0.86	0.80	0.88	0.80	0.96	0.81
	**95% CI**	[0.33, 0.96]	[0.02, 0.96]	[0.66, 0.98]	[0.42, 0.96]	[0.12, 0.96]	[0.51, 0.97]	[0.12, 0.96]	[0.85, 0.99]	[0.16, 0.96]

* Indicates a statistical difference between the force plate and loadsol^®^ value.

**Table 4:** Between-day reliability ICC and SEM values with the 95% confidence intervals.

Speed			3.5 m/s			3.0 m/s			1.3 m/s		
% Grade			0%	−10%	10%	0%	−10%	10%	0%	−10%	10%
**Peak Force**	**FP**	**ICC**	0.97	0.96	0.93	0.97	0.96	0.96	0.89	0.97	0.93
		**95% CI**	[0.94, 0.99]	[0.92, 0.98]	[0.84, 0.97]	[0.94, 0.99]	[0.91, 0.98]	[0.92, 0.98]	[0.98, 1.00]	[0.96, 0.99]	[0.94, 0.99]
	**LS**	**ICC**	0.89	0.88	0.93	0.91	0.90	0.91	0.96	0.97	0.95
		**95% CI**	[0.77,0.95]	[0.73,0.94]	[0.84,0.96]	[0.81,0.96]	[0.79,0.96]	[0.82,0.96]	[0.91,0.98]	[0.93,0.99]	[0.88,0.98]
**Impulse**	**FP**	**ICC**	0.80	0.92	0.81	0.74	0.89	0.88	0.91	0.97	0.86
		**95% CI**	[0.57,0.91]	[0.82,0.96]	[0.60,0.91]	[0.43,0.88]	[0.76,0.95]	[0.75,0.95]	[0.98,1.00]	[0.93,0.99]	[0.94,0.99]
	**LS**	**ICC**	0.91	0.90	0.88	0.88	0.92	0.86	0.96	0.96	0.95
		**95% CI**	[0.81,0.96]	[0.78,0.95]	[0.73,0.94]	[0.74,0.94]	[0.84,0.96]	[0.70,0.93]	[0.91,0.98]	[0.92,0.98]	[0.88,0.98]
**Loading Rate**	**FP**	**ICC**	0.88	0.91	0.87	0.70	0.81	0.92	0.91	0.91	0.94
		**95% CI**	[0.75,0.95]	[0.79,0.96]	[0.72,0.94]	[0.43,0.86]	[0.59,0.91]	[0.83,0.96]	[0.96,0.91]	[0.88,0.98]	[0.81,0.97]
	**LS**	**ICC**	0.88	0.76	0.92	0.85	0.74	0.90	0.90	0.96	0.72
		**95% CI**	[0.74, 0.95]	[0.44, 0.90]	[0.82, 0.96]	[0.67, 0.93]	[0.44, 0.88]	[0.79, 0.95]	[0.78, 0.96]	[0.92, 0.98]	[0.35, 0.88]
